# Factors associated with improvement in disability-adjusted life years in patients with HIV/AIDS

**DOI:** 10.1186/1471-2458-8-362

**Published:** 2008-10-21

**Authors:** Clara Bermudez-Tamayo, Jose Jesus Martin Martin, Isabel Ruiz-Pérez, Antonio Olry de Labry Lima

**Affiliations:** 1 Andalusian School of Public Health, Granada, Spain; 2 Department of Applied Economics, University of Granada, Granada, Spain; 3 Andalusian School of Public Health, CIBER de Epidemiología y Salud Pública (CIBERESP), Granada, Spain

## Abstract

**Background:**

The epidemic of HIV/AIDS and treatments that have emerged to alleviate, have brought about a shift in the burden of disease from death to quality of life/disability. The aim was to determine which factors are associated with improvements in the level of health of male and female patients with HIV/AIDS in Andalusia, in terms of disability-adjusted life years.

**Methods:**

Descriptive study based on a sample group of 8800 people on the Andalusian AIDS register between 1983 and 2004. Dependent variables: Life lost due to premature mortality (YLL), years lost due to disability (YLD) and disability-adjusted life years (DALY). Independent variables: vital state, sex, age at the time of diagnosis, age at the time of death, transmission category, province of residence, AIDS-indicator disease and the period of diagnosis. A bivariate analysis was carried out to find out if the health level variables changed in accordance with the independent variables. Using the independent variables which had a statistically significant link with the level of health variables, a multivariate linear regression model, disaggregated by gender, was constructed.

**Results:**

Amongst the women, we found a model which explained the level of health of 64.9%: a link was found between a higher level of health (lower DALYs) and not intravenous drug use, the province of residence, being diagnosed during the HAART era and older age at the time of diagnosis. Amongst the men, we found a model which explained the level of health of 64.4%: a link was found between a higher level of health (lower DALYs) and intravenous drug use, the province of residence, being diagnosed during the HAART era and older age at the time of diagnosis.

**Conclusion:**

A higher level of health (lower DALY) amongst both men and women was found to be linked to not be intravenous drug user, the province of residence, being diagnosed during the HAART era and older age at the time of diagnosis.

## Background

The human immunodeficiency virus (HIV) and acquired autoimmune deficiency syndrome (AIDS) epidemic constitutes a large-scale worldwide public health problem, with important demographic, social and economic repercussions. HIV/AIDS has resulted in the deaths of more than 25 million people since it was first identified in 1981. This means that it is one of the most destructive epidemics in living history [1].

HIV/AIDS has been classified as a chronic disease [2]. The WHO uses this term for diseases which last a long duration, there are many opportunities for prevention and they require a long-term and systematic approach to treatment. It is therefore very important to find out about the different ways in which HIV affects mortality rates within the infected population and to identify the factors associated with better levels of health. By reading the literature, we can see that the most important factors studied which can be linked to lower health scores are sex, level of education or socioeconomic level, the virus transmission category, age and geographical location.

In terms of AIDS incidence and mortality rates, previous studies have shown that there are differences between men and women, although these differences are not always the same in all cases. In Spain, incidence and mortality rates are different, in accordance with these trends [3].

The most common transmission method in Spain has been through the use of intravenous drugs. Many different studies have shown the differences in the advance of the disease amongst intravenous drug users in comparison with patients who contracted the disease in other ways. Patients who use drugs tend to have a poorer prognosis. Although these differences also vary between regions [4,5].

With regard to differences between regions, there is great variation in less developed countries such as Brazil. Brito [6] examined the characteristics of the AIDS epidemic between regions and found that, from 1996 onwards, the incidence rate of AIDS amongst adults in Brazil and Sao Paulo was reaching a stable plateau, while in northwest Brazil incidence rates of the illness continued to increase. In addition, Szwarcwald [7] found that AIDS incidence rates were higher around the port of Rio de Janeiro. She also found that the number of cases amongst women increased during the final phase of the study (post-HAART), with higher incidence rates in the poorest areas. In Europe, analyses of large areas have revealed that there is not much variation. Van Astern [8] studied HIV/AIDS mortality rates in European countries and the results showed that there was little geographical variation. However, analyses of smaller areas have revealed some variation. For example, Giovannetti [9] found differences in AIDS mortality rates between residents of coastal areas and other provinces in Italy both before and after the introduction of HAART, and concluded that the variations may reflect differences in the composition of the groups and access to treatment. Spain has a comprehensive health system of universal coverage, and offers HAART as one possible treatment. It is therefore unlikely that there would be any significant intra-territorial variations.

In terms of levels of education and socioeconomic levels, studies have shown that, in a number of different settings, patients with a higher level of education have a better prognosis [6,10-12] and a higher medication adherence rate [6].

Specialist studies have shown that physical quality of life decreases as HIV progresses, as a result of symptoms and antiretroviral treatment [13]. As such, scores to measure patients' level of health, such as Disability-Adjusted Life Years (DALY), are more appropriate than mortality or survival rates when it comes to studying the impact of this illness or the associated "burden of disease", as they provide more information, taking into account not only that patients live for longer, but that their quality of life is also better. A patient's DALY represents the current value of the number of years of life free from disability that he or she will live, which are lost or gained as a result of premature death or disability during a certain year [14].

The aim of this study is to determine which factors are associated with improvements to the level of health of male and female patients with HIV/AIDS in Andalusia, in terms of disability-adjusted life years.

## Methods

### Design

Descriptive study. 9699 people included on the Andalusia AIDS Register between 1983 and 2004 were considered for the study. 246 were excluded because there was no date of death, 482 because there was no date of diagnosis and a further 171 because their place of residence was outside Andalusia. The final population taken into account for this study was 8800 people.

The data used for this study were extracted from the Andalusian Register of AIDS cases, which forms part of the national system of epidemiological monitoring in which all 17 of the Spanish Autonomous Communities participate.

### Variables

#### Dependent Variables

##### Disability-Adjusted Life Years

Disability-adjusted life years can be calculated using the following equation:

DALY = YLL + YLD

YLL = Years of life lost due to premature mortality

YLD = Years lost due to disability

###### Life lost due to premature mortality (YLL)

The YLL is an estimated figure reflecting the number of years lost as a result of premature death based on a predetermined life expectancy. The YLL is calculated based on the life expectancy at the age of death obtained via a standard life table with a low mortality [15].

###### Years lost due to disability (YLD)

The YLD is an estimate of the number of years that a patient has lived with a disability [15]. *YLD *= N* *D*

N_i _= Duration of (years lived) with the illness for the patient i.

D = Value reflecting disability between 0 = maximum health and 1 = death.

For the purposes of this study, we have used the results of research carried out by [16] with the European Disability Weights Group, which assigns Spain a value of 0.65 for HIV/AIDS for this indicator.

#### Independent Variables

##### Vital State

This is the information collected regarding the vital state of HIV/AIDS patients on the 31^st ^of May 2005. There are two possible categories: alive or dead.

##### Sex

Two possible categories: male or female.

##### Age at the Time of Diagnosis

A continuous variable calculated based on the patient's date of diagnosis and date of birth.

##### Age at Time of Death

A continuous variable calculated based on the patient's date of death and date of birth.

##### Transmission Category

This variable reflects the reason why the illness was transmitted, taking into account whether or not the individual belongs to a risk group of some sort. This variable has four categories: homosexual, heterosexual, intravenous drug user and other.

• The "homosexuals" category refers to homosexual individuals who engage in sexual relations entailing risk, i.e. without using a condom.

• The "heterosexuals" category refers to heterosexual individuals who engage in sexual relations entailing risk, i.e. without using a condom.

• The "other" category includes categories with few cases, such as recipients of blood products, children of at-risk mothers, patients infected by blood transfusions, and unknown cause.

##### Province of Residence

Province of residence of the patient at the time of diagnosis.

##### Diagnosis Period

Three diagnosis periods have been used based on the level of implementation of HAART in Spain: pre-HAART (before 1996), limited use of HAART (1997 and 1998) and HAART (after 1998).

### Analysis

A bivariate analysis was carried out to find out if the health level variables changed in accordance with the independent variables (DALY, YLL and YLD). The inclusion of these variables is justified according to previous studies detailed in the literature. The Student's t-test and ANOVA table were used to examine the qualitative variables with variance homogeneity and normal distribution, and the other variables were analysed using the Mann-Whitney U-test and the Kruskall Wallis H-test. The continuous independent variables were examined using linear regression analysis. All of the analyses were carried out independently for the men and women in the sample group.

Using the independent variables which proved to have a statistically significant link with the DALY variable, we constructed two multivariate linear regression models – one for the men and one for the women.

*Healthlevel*_*i *_= *β*_0 _+ *β*_1_*R*_*i *_+ *β*_2_*D*_*i *_+ *β*_3_*I*_*i *_+ *β*_4_*A*_*i*_

where *Health level*_*i *_is the dependent variable of the (DALY) level for a person with HIV/AIDS *i*

*R*_*i*_: Route of infection

*D*_*i*_: Place of residence

*I*_*i*_: Period of diagnosis

*A*_*i *_Age at the time of diagnosis

Todos los análisis fueron efectuados con el software SPSS for Windows 14.0.

The research commission of the institution granted ethical approval for the study.

## Results

Table 1 provides basic data about patients infected with HIV/AIDS in Andalusia. Of all the subjects included in the study, 7375 were men (83.8%). 60.9% of the subjects died during the study period, 51.4% of the women and 62.7% of the men. The most frequent route of HIV infection was through intravenous drug use (56.28 for Women and 74.01 for men).

**Table 1:** Descriptive Analysis of the Population Studied.

**VARIABLE**	**Total**		**Women**		**Men**	
	**n**	**%**	**n**	**%**	**n**	**%**
**Deceased**						
No	3440	39.1	693	48.63	2747	37.25
Yes	5360	60.9	732	51.37	4628	62.75
**Route of Infection**						
IVDU	6260	71.1	802	56.28	5458	74.01
Heterosexual	1174	13.3	468	32.84	706	9.57
Homosexual	763	8.7	0	0.00	763	10.35
Other	492	5.6	149	10.46	343	4.65
**Province of residence at diagnosis**						
Jaen	444	5.0	81	5.68	363	4.92
Huelva	511	5.8	81	5.68	430	5.83
Almeria	600	6.8	124	8.70	476	6.45
Cordoba	608	6.9	109	7.65	499	6.77
Granada	871	9.9	158	11.09	713	9.67
Cadiz	1541	17.5	254	17.82	1287	17.45
Seville	1713	19.5	215	15.09	1498	20.31
Malaga	2511	28.5	403	28.28	2108	28.58

Qualitative variables

IVDU: Intravenous drug users

With regard to the province of origin of patients with HIV/AIDS, the province with the least cases in Andalusia was Jaen (444), followed by Huelva and Almeria, with 511 and 600 cases respectively. The provinces with the most cases were Cadiz (1541), Seville (1713) and Malaga (1511).

The average age for AIDS diagnosis was 33.7 years of age. The average age at the time of diagnosis was 31.3 years for women (SD 10.5) and 34.1 for men (SD 9.0). The average survival time for those infected with HIV/AIDS was 49.6 months (SD 50.4) and the average age at time of death was 36.3 for women (SD 10.5) and 38.6 for men (SD 9.3) (Table 2).

**Table 2:** Descriptive Analysis of the Population Studied.

	**Total**		**Women**		**Men**	
**Variable**	**Mean (Stand. Dev.)**	**Range**	**Mean (Stand. Dev.)**	**Range**	**Mean (Stand. Dev.)**	**Range**
Age at diagnosis N = 8800	33.7 (9.31)	0.0–87	31.3 (10.5)	0.0–77.0	34.1 (9.0)	0.0–87.0
Survival in months N = 8800	49.6 (50.6)	0.1–236.4	54.7 (51.8)	0.1–234.4	48.7 (50.4)	0.0–236.4
Age At Time of Death. N = 5389	38.2 (9.5)	0.2–87.6	36.3 (10.5)	0.2–77.9	38.6 (9.3)	0.3–87.7
Years of Life Lost. N = 8796	29.06 (24.36)	0.0–82.50	26.82 (27.09)	0–82.5	29.49 (23.77)	0–80
Years of Life with Disability. N = 8800	3.95 (4.06)	0.0–19.18	4.33 (4.16)	0.01–19.01	3.88 (4.04)	0.00–19.18
DALY. N = 8796	33.01 (21.98)	0.08–85.96	31.15 (24.78)	0.08–85.96	33.37 (21.38)	0.08–83.11

Quantitative variables

DALY: Disability-adjusted life years

In terms of the level of health, the average DALY for patients with HIV/AIDS in Andalusia was 33.01 (SD 21.98) – 31.15 for the women (SD 24.78) and 33.37 for the men (SD 21.38). The average number of Years of Life Lost due to HIV/AIDS was 29.06 (SD 24.36) – 26.82 for the women (SD 27.09) and 29.49 for the men (23.77). The average value of Years Lived with Disability due to HIV/AIDS was 3.95 (SD 4.06) – 4.33 for the women (SD 4.16) and 3.88 for the men (4.04).

### Variables Associated with Improved Levels of Health

#### Model for the Women

In the bivariate analysis, female intravenous drug users had lower levels of health, i.e. they had higher YLLs, YLDs and DALYs due to HIV/AIDS (YLL = 29.4, YLD = 4.58 and DALY = 34.03) than those who did not use intravenous drugs (YLL = 23.4, YLD = 3.99 and DALY = 27.43) (Table 3).

**Table 3:** Bivariate Analysis for Women.

**VARIABLE**	**YLL**		**YLD**		**DALY**	
	**Mean (Stand. Dev.)**	**p-value**	**Mean (Stand. Dev.)**	**p-value**	**Mean (Stand. Dev.)**	**p-value**
**IVDU**						
No	23.44 (27.34)	< 0.001	3.99 (3.88)	0.009	27.43 (25.39)	< 0.001
Yes	29.46 (26.64)		4.58 (4.33)		34.03 (23.97)	
**Province of residence at diagnosis**						
Jaen	22.44 (26.25)	0.126	4.78 (4.44)	0.347	27.22 (23.61)	0.027
Huelva	30.22 (27.35)	0.252	4.51 (4.39)	0.704	34.73 (24.88)	0.186
Almeria	26.97 (27.09)	0.948	4.44 (3.63)	0.723	31.42 (24.92)	0.902
Cordoba	32.06 (26.84)	0.036	3.97 (4.60)	0.393	36.02 (23.96)	0.039
Granada	18.58 (25.27)	< 0.001	5.06 (4.10)	0.020	23.63 (23.43)	< 0.001
Cadiz	29.48 (27.02)	0.084	4.10 (4.24)	0.331	33.58 (24.63)	0.084
Seville	25.14 (27.67)	0.333	4.75 (4.14)	0.111	29.89 (25.24)	0.424
Malaga	28.00 (27.04)	0.300	3.91 (4.02)	0.014	31.91 (24.94)	0.469
**Diagnosis Period**						
Up to and including 1996	35.89 (26.40)		4.92 (4.89)		40.81 (22.47)	
1997 and 1998	16.57 (24.35)	< 0.001	5.47 (2.77)	0.127	22.04 (21.97)	< 0.001
1999 onwards	12.77 (21.48)	< 0.001	2.53 (1.90)	< 0.001	15.31 (20.54)	< 0.001

Qualitative Variables

IVDU: Intravenous drug users

YLL: Life lost due to premature mortality

YLD: Years lost due to disability

DALY: Disability-adjusted life years

In terms of levels of health and the place of residence at the time of diagnosis, a link was found between being a resident of Cordoba and higher Years of Life Lost (p = 0.036) and higher DALYs (p = 0.039). Likewise, a link was found between living in Malaga and having a lower YLD.

In addition, a link was found between living in Granada at the time of diagnosis and lower YLLs (p < 0.001), higher YLDs (p = 0.020) and lower DALYs (p < 0.001).

A link was found between older age at the time of diagnosis and lower Years of Life Lost due to the illness (Coefficient = -0.446), lower Years of Life with Disability (Coefficient = -0.087) and lower Disability-Adjusted Life Years (Coefficient = -0.533).

In the multivariate analysis with linear regression a model was found which explained 24.5% (Coefficient of determination = 0.245) of the level of health (DALY) (Table 4). A higher level of health (lower DALY) was found to be linked to intravenous drug use, the province of residence, being diagnosed during the HAART era and older age at the time of diagnosis.

**Table 4:** Multivariate Analysis for Women.

**VARIABLE**	**Constant**	**Standard error**	**p-value**
Constant	44.775	2.609	< 0.001
**IVDU**			
No			
Yes	2.692	1.181	0.023
**Province of residence at diagnosis**			
Granada			
Almeria	2.512	2.616	0.337
Cadiz	4.829	2.213	0.029
Cordoba	5.681	2.725	0.037
Huelva	4.209	2.976	0.158
Jaen	0.429	2.972	0.885
Malaga	3.801	2.044	0.063
Seville	0.784	2.286	0.732
**Period of diagnosis**			
Up to and including 1996			
1997 and 1998	-17.458	1.745	< 0.001
1999 onwards	-22.834	1.396	< 0.001
**Age at diagnosis**	-0.298	.056	< 0.001

Coefficient of determination = 24.5%

IVDU: Intravenous drug users

Dependent variable: DALY

#### Model for the Men

In the bivariate analysis, male intravenous drug users had lower levels of health, i.e. they had higher YLLs, YLDs and DALYs due to HIV/AIDS (YLL = 31.19, YLD = 4.01 and DALY = 35.19) than those who did not use intravenous drugs (YLL = 23.78, YLD = 3.52 and DALY = 27.30) (Table 5).

**Table 5:** Bivariate Analysis for Men.

**VARIABLE**	**YLL**		**YLD**		**DALY**	
	**Mean (Stand. Dev.)**	**p-value**	**Mean (Stand. Dev.)**	**p-value**	**Mean (Stand. Dev.)**	**p-value**
**IVDU**						
No	23.78 (22.49)	< 0.001	3.52 (3.90)	< 0.001	27.30 (20.43)	< 0.001
Yes	31.19 (23.86)		4.01 (4.06)		35.19 (21.31)	
**Province of residence at diagnosis**						
Jaen	27.59 (25.05)	0.137	3.86 (3.90)	0.928	31.45 (22.65)	0.097
Huelva	31.78 (23.31)	0.039	3.66 (3.99)	0.241	35.44 (20.91)	0.039
Almeria	31.16 (23.50)	0.109	3.51 (3.82)	0.030	34.68 (21.32)	0.168
Cordoba	30.80 (23.97)	0.207	3.81 (4.61)	0.723	34.62 (21.08)	0.173
Granada	26.85 (24.17)	0.002	3.88 (3.95)	0.097	30.73 (22.07)	0.001
Cadiz	31.28 (23.29)	0.003	3.85 (3.96)	0.769	35.13 (20.86)	0.001
Seville	25.14 (27.67)	0.333	4.75 (4.14)	0.111	29.89 (25.24)	0.424
Malaga	30.24 (23.36)	0.182	3.69 (3.97)	0.010	33.93 (21.08)	0.150
**Diagnosis Period**						
Up to and including 1996	36.28 (21.97)		4.22 (4.61)		40.50 (18.33)	
1997 and 1998	20.81 (22.91)	< 0.001	4.67 (3.03)	0.134	25.48 (20.36)	< 0.001
1999 onwards	15.18 (21.06)	< 0.001	2.42 (1.91)	< 0.001	17.60 (20.03)	< 0.001

Qualitative Variables

In terms of levels of health and the place of residence at the time of diagnosis, living in Huelva (p = 0.039) and Cadiz (p = 0.003) was found to be linked to higher YLLs and higher DALYs (p = 0.039), living in Almeria (p = 0.030) and Malaga (p = 0.010) was to be linked to higher YLDs, and living in Huelva (p = 0.039) and Cadiz (p = 0.001) was found to be linked to higher DALYs. In addition, a link was found between living in Granada at the time of diagnosis and lower DALYs (p = 0.001).

Older age at the time of diagnosis was found to be linked to lower Years of Life Lost due to the illness (Coefficient = -20.437), lower Years of Life with Disability (Coefficient = -20.17) and lower Disability-Adjusted Life Years (Coefficient = -16.411).

In the multivariate analysis with linear regression a model was found which explained 64.4% (Coefficient of determination = 0.644) of the level of health (DALY). A higher level of health (lower DALY) was found to be linked to intravenous drug use, the province of residence, being diagnosed during the HAART era and older age at the time of diagnosis (Table 6).

**Table 6:** Multivariate Analysis for Men.

**VARIABLE**	**Constant**	**Standard error**	**p-value**
Constant	52.005	1.331	< 0.001
**IVDU**			
No	1		
Yes	2.857	0.545	< 0.001
**Province of residence at diagnosis**			
Granada	1		
Almeria	1.886	1.100	0.086
Cadiz	0.585	0.873	0.503
Cordoba	0.045	1.088	0.967
Huelva	2.228	1.132	0.049
Jaen	-1.332	1.204	0.268
Malaga	0.460	0.810	0.570
Seville	-2.221	0.848	0.009
**Period of diagnosis**			
Up to and including 1996	1		
1997 and 1998	-13.530	0.651	< 0.001
1999 onwards	-19.954	0.556	< 0.001
**Age at diagnosis**	-0.427	0.027	< 0.001

Coefficient of determination = 64.4%

IVDU: Intravenous drug users

Dependent variable: DALY

## Discussion

The most innovative part of this study is the use of DALYs to measure the level of health of patients in the sample group. DALYs are a more holistic measurement than mortality or survival rates to study the impact or "burden of disease" associated with HIV/AIDS, because, as we know, quality of life decreases as the disease progresses as a result of its symptoms [13].

This study also contributes to knowledge of this area by tackling the models separately for each sex, as there is some debate surrounding the possible differences in factors which explain health scores between men and women with HIV/AIDS. Based on biological factors, the first studies carried out regarding the differences in HIV prognosis between men and women found that the disease progressed more slowly in women than men, so female patients died after a longer period. However, later studies have come to different conclusions, attributing the differences in prognosis to other factors [17]. In this study, we have found that the factors related to levels of health were the same for both sexes, although the relative risks were different.

The prevalence of AIDS in Andalusia is lower than the Spanish average, with 1.23 cases per 1000 inhabitants [18], and it is the Spanish Autonomous Community with the lowest percentage of female patients with HIV/AIDS.

This study found that the use of intravenous drugs was related to lower levels of health. Intravenous drug use is the most common means of transmission of HIV/AIDS in Spain [3]. This link varies according to different factors, as stated in the literature. Some studies have found differences between intravenous drug use and other routes of infection in the mortality and survival rates in different periods, both in Spain and in other European countries [4,5,8]. However, some other studies do not reflect the same results [19].

As HIV positive intravenous drug users lack information and are excluded and discriminated against [1], sometimes rates of adherence to antiretroviral treatment are "unacceptably low" amongst drug users. In addition, as intravenous drug users have a particular sociocultural environment and have to deal with drug addiction as well as their HIV infection, it is difficult to monitor these patients and for them to adhere correctly to their treatment programmes, and this compromises the effectiveness of treatment and results in resistance to it [20], thus affecting quality of life and levels of health.

A link was found between diagnosis after the introduction of HAART (1997 and 1998, 1999 onwards) and a higher level of health amongst patients with HIV/AIDS. This reflects the effectiveness of the treatment. Most studies have shown that HAART is more effective than the technologies used before its introduction [21-24].

The differences in levels of health according to the province of residence at the time of diagnosis may be a result of different clinical practices in the different healthcare centres. It is also possible that different cultural factors influence patients' access to healthcare services. Other studies must be carried out in order to evaluate these differences.

An older age at time of diagnosis linked to lower DALY, it could be related to shorter life expectancy. This result could be used as an argument in the debate about whether it is appropriate to assign age-related weightings in the calculation of DALYs. However, some researchers believe that if age is taken into account, other factors such as social roles (occupation or productivity), or income, would also have to be considered [25]. The main problem is whether a DALY (or a QALY) has the same value for all patients. This equality debate centres around a utilitarian philosophy and is, of course, a matter of personal opinion. However, any other alternative would make it necessary to specify which criteria mean that a DALY for a child or an elderly person is more or less valuable than that of another person.

The factors linked to the level of health were the same for both sexes. However, the coefficient of determination was different, and was higher amongst the men in the sample group than amongst the women. This suggests that there are other variables at play which are not specified in the model (not available in the database), and which lead to differences and asymmetry between the two groups. The data merely allow us to make speculative suggestions about what these other variables may be, and they could include access to the healthcare system, social support and even therapeutic bias.

One limiting factor of this study is the fact that it did not include clinical factors, such as CD4 levels, the viral load or severity of the illness, number of years as an HIV carrier, adherence to treatment or coinfection, and as such the mortality rate was not adjusted to take these parameters into account. We would recommend that these data are included in future research so that the results of this study can be confirmed.

Advances in HIV/AIDS treatment have led to greater survival rates and limited quality of life amongst patients, with a considerable burden of disease. Healthcare services must meet the challenge of offering the care required for HIV/AIDS patients, providing them with the treatment available and facilitating access to the whole population. In this way, factors which are extrinsic to the patient's clinical situation, such as the province of residence at the time of diagnosis or intravenous drug use, will cease to be related to the burden of disease. However, other socio-economic attributes could have played important role independent of treatment and care, more so when injecting drug users constituted the bulk of the study populations. Improvement of HIV care would certainly improve DALY but not completely because HIV is not simply a medical disease.

## Conclusion

A higher level of health (lower DALY) amongst both men and women was found to be linked to intravenous drug use, the province of residence, being diagnosed during the HAART era and older age at the time of diagnosis.

## List of abbreviations

HIV/AIDS: The human immunodeficiency virus (HIV) and acquired autoimmune deficiency syndrome (AIDS); YLL: Life lost due to premature mortality; YLD: Years lost due to disability; DALY: Disability-adjusted life years.

## Competing interests

The authors declare that they have no competing interests.

## Authors' contributions

CBT and AOLL drafted the manuscript. CBT participated in the design of the study and performed the statistical analysis. JJMM and IRP conceived of the study, participated in its design and coordination and helped to draft the manuscript. All authors read and approved the final manuscript.

## Pre-publication history

The pre-publication history for this paper can be accessed here:



## References

[B1] Report on the Global AIDS Epidemic. http://www.unaids.org/en/KnowledgeCentre/HIVData/GlobalReport/2006/default.asp.

[B2] Chronic Disease. An Economic Perspective. Consulted on 7-07-2007. http://www.oxha.org/initiatives/economics/chronic-disease-an-economic-perspective.

[B3] National Register of Cases of AIDS. Consulted on 29-09-2007. http://www.isciii.es/htdocs/pdf/informe_sida.pdf.

[B4] Pérez-Hoyos S, del Amo J, Muga R, del Romero J, García de Olalla P, Guerrero R, Hernàndez-Aguado I, GEMES (Spanish Multicenter Study Group of Seroconverters) (2003). Effectiveness of highly active antiretroviral therapy in Spanish cohorts of HIV seroconverters. differences by transmission category. AIDS.

[B5] Porter K, Babiker A, Bhaskaran K, Darbyshire J, Pezzotti P, Porter K, Walker AS, CASCADE Collaboration (2003). Determinants of survival following HIV-1 seroconversion after the introduction of HAART. Lancet.

[B6] Brito AM, Castilho EA, Szwarcwald CL (2005). Regional patterns of the temporal evolution of the AIDS epidemic in Brazil following the introduction of antiretroviral therapy. Braz J Infect Dis.

[B7] Szwarcwald CL, Bastos FI, Barcellos C, Esteves MA, Castilho EA (2001). AIDS epidemic dynamics in the municipality of Rio de Janeiro, Brazil, 1988–1996. Spatial-temporal statistic modelling. Cad Saude Publica.

[B8] Van Asten L, Zangerle R, Hernandez Aguado I, Boufassa F, Broers B, Brettle RP, Roy Robertson J, McMenamin J, Coutinho RA, Prins M (2005). Do HIV disease progression and HAART response vary among injecting drug users in Europe?. Eur J Epidemio.

[B9] Giovannetti L, Crocetti E, Chellini E, Martini A, Balocchini E, Costantini AS (2004). Temporal trends in AIDS incidence and mortality in Tuscany (1987–2000)]. Epidemiol Prev.

[B10] Jarrin I, Lumbreras B, Ferreros I, Pérez-Hoyos S, Hurtado I, Hernández-Aguado I (2007). Effect of education on overall and cause-specific mortality in injecting drug users, according to HIV and introduction of HAART. Int J Epidemiol.

[B11] Báez-Feliciano DV, Thomas JC, Gómez MA, Miranda S, Fernández DM, Velázquez M, Ríos-Olivares E, Hunter-Mellado RF (2005). Changes in the AIDS epidemiologic situation in Puerto Rico following health care reform and the introduction of HAART. Rev Panam Salud Publica.

[B12] del Amo J, del Romero J, Barrasa A, Pérez-Hoyos S, Rodríguez C, Díez M, García S, Soriano V, Castilla J, Grupo de Seroconvertores de la Comunidad de Madrid (2002). Factors influencing HIV progression in a seroconverter cohort in Madrid from 1985 to 1999. Sex Transm Infect.

[B13] Ruiz-Pérez I, Olry de Labry-Lima A, López-Ruz MA, del Arco-Jiménez A, Rodríguez-Baño J, Causse-Prados M, Pasquau-Liaño J, Martín-Rico P, Prada-Pardal JL, de la Torre-Lima J, López-Gómez M, Marcos M, Muñoz N, Morales D, Muñoz I (2005). Estado clínico, adherencia al TARGA y calidad de vida en pacientes con infección por el VIH tratados con antirretrovirales. Enferm Infecc Microbiol Clin.

[B14] Gold MR, Stevenson D, Fryback DG (2002). HALYS and QALYS and DALYS, Oh My: similarities and differences in summary measures of population Health. Annu Rev Public Health.

[B15] Mathers CD, Murray CJL, Ezzati M, Gakidou E, Salomon JA, Stein C (2000). Population health metrics: crucial inputs to the development of evidence for health policy. Population Health Metrics.

[B16] Schwarzinger M, Stouthard M, Burström K, Nord E, the European Disability Weights Group (2003). Cross-national agreement on disability weights. the European Disability Weights Project. Population Health Metrics.

[B17] Shah R, Bradbeer C (2000). Women and HIV – revisited ten years on. Int J STD AIDS.

[B18] Bermúdez MP, Teva I (2004). Situación Actual del SIDA en España: Análisis de las diferencias entre comunidades Autónomas. International Journal of Clinical and Health Psychology.

[B19] Mocroft A, Gill MJ, Davidson W, Phillips AN (2000). Are there gender differences in starting protease inhibitors, HAART, and disease progression despite equal access to care?. J Acquir Immune Defic Syndr.

[B20] Tornero C, Santamaria S, Gil E (2004). Distribución del gasto farmacéutico en medicación antirretoviral. An Med Interna.

[B21] Messori A, Trippoli S, Vaiani M (2001). The cost effectiveness of antiretroviral therapy for HIV disease. N Engl J Med.

[B22] Anis AH, Hogg RS, Wang XH, Yip B, Palepu A, Montaner JS, O'Shaughnessy MV, Schechter MT (1998). Modelling the potential economic impact of viral load-driven triple drug combination antiretroviral therapy. Pharmacoeconomics.

[B23] Miners AH, Sabin CA, Trueman P, Youle M, Mocroft A, Johnson M, Beck EJ (2001). Assessing the cost-effectiveness of HAART for adults with HIV in England. HIV Med.

[B24] Freedberg KA, Losina E, Weinstein MC, Paltiel AD, Cohen CJ, Seage GR, Craven DE, Zhang H, Kimmel AD, Goldie SJ (2001). The cost effectiveness of combination antiretroviral therapy for HIV disease. N Engl J Med.

[B25] Anand D, Hanson K (1997). Disability-adjusted life years: a critical review. J Health econ.

